# Demographic consequences of an extreme heat wave are mitigated by spatial heterogeneity in an annual monkeyflower

**DOI:** 10.1002/ece3.10397

**Published:** 2023-08-09

**Authors:** Laura M. McDonald, Anna Scharnagl, Andrea K. Turcu, Courtney M. Patterson, Nicholas J. Kooyers

**Affiliations:** ^1^ Department of Biology University of Louisiana Lafayette Louisiana USA; ^2^ Department of Integrative Biology University of California Berkeley California USA

**Keywords:** adaptation lag, common garden experiment, demography, evolutionary ecology, global change ecology, *Mimulus guttatus* (*Erythranthe guttata*), natural selection, phenology

## Abstract

Heat waves are becoming more frequent and intense with climate change, but the demographic and evolutionary consequences of heat waves are rarely investigated in herbaceous plant species. We examine the consequences of a short but extreme heat wave in Oregon populations of the common yellow monkeyflower (*Mimulus guttatus*) by leveraging a common garden experiment planted with range‐wide populations and observational studies of 11 local populations. In the common garden, 89% of seedlings died during the heat wave including >96% of seedlings from geographically local populations. Some populations from hotter and drier environments had higher fitness, however, others from comparable environments performed poorly. Observational studies of local natural populations drastically differed in the consequences of the heat wave—one population was completely extirpated and nearly half had a >50% decrease in fitness. However, a few populations had *greater* fitness during the heat wave year. Differences in mortality corresponded to the impact of the heat wave on soil moisture—retention of soil moisture throughout the heat wave led to greater survivorship. Our results suggest that not all populations experience the same intensity or degree of mortality during extreme events and such heterogeneity could be important for genetic rescue or to facilitate the distribution of adaptive variants throughout the region.

## Graphical abstract

We demonstrate that although heat waves may be devastating for certain populations in a region, not all populations experience the same intensity or mortality. This heterogeneity in selective pressures within and among populations may be important for genetic rescue or to facilitate the distribution of adaptive variants throughout the region.

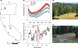

## 1 INTRODUCTION

Climate change is not only causing gradual increases in global mean temperatures but is also causing higher levels of variation in temperature and precipitation in specific areas across the world (Pachauri et al., 2014). This increase in variation suggests extreme climatic events, such as droughts, floods, and heat waves, will become more common and more severe in certain locations (Dai, 2013; Guerreiro et al., 2018; Pachauri et al., 2014; Scherrer et al., 2016). While there is often time for species to disperse to areas with more optimal conditions during prolonged extreme events (Tingley et al., 2009), pulses of extreme climate conditions can be challenging for organisms with limited movement, potentially causing severe mortality with lasting demographic consequences or even extirpation (Jiménez et al., 2011; Ruthrof et al., 2018; Sumerford et al., 2000). Such extreme climatic events in natural populations are challenging to study because these events are rare by definition, occur unpredictably, are short‐lived, and often require background information or data on a specific species collected before the event to address meaningful biological questions (Gutschick & BassiriRad, 2003).

Heat waves, defined as three or more consecutive days where the temperature is greater than the 90th percentile for a given location and time of year (Perkins & Alexander, 2013), have increased dramatically over the last century (Coumou et al., 2013; Della‐Marta et al., 2007) and are predicted to further increase in frequency, duration, and intensity in the coming century (Coumou et al., 2013; Meehl & Tebaldi, 2004; Perkins‐Kirkpatrick & Gibson, 2017). Such heat waves and associated water availability stress have been linked to mass mortality events in natural populations (Breshears et al., 2005; Matusick et al., 2018; Ruthrof et al., 2018) and substantial loss of yield or even complete crop failure in agricultural systems (Zampieri et al., 2017). However, there are relatively few studies of heat waves documented in natural populations, especially in herbaceous plant populations, that examine how these events impact immediate population fitness and long‐term population dynamics (but see Sheth and Angert (2018), Thomson et al. (2018), Harrison and LaForgia (2019)). These data are critical for determining extirpation risks for populations and future evolutionary responses (Kooyers et al., 2021).

While native populations may struggle with extreme conditions caused by heat waves, geographically distant populations that have historically experienced hotter and/or drier conditions may be better adapted to such conditions. Indeed, experimental studies from crops and model systems including *Oryza*, *Zea*, and *Arabidopsis* indicate that there is substantial genetic variation in escape, avoidance, and tolerance to heat stress within species (Janni et al., 2020; Silva‐Correia et al., 2014) and populations that experience heat stress more often are better adapted to it (Shah et al., 2011; VanWallendael et al., 2019). Additionally, adaptation lags, where geographically distant populations are better adapted than the native population to a site because of shifting climate, have been observed frequently in the context of variation in annual climates rather than in extreme short‐term climatic events (Anderson & Wadgymar, 2020; Kooyers et al., 2019; Wilczek et al., 2014).

Alternatively, there are a number of reasons why populations from hotter and drier regions may *not* produce a fitness advantage over native populations during a heat wave. Historically, hotter and drier populations may not have evolved resistance mechanisms sufficient to withstand extreme and rapid heat waves. That is, optimal physiological performance at a higher temperature is not necessarily the same agent of selection as survival during short‐term heat shock or performance within strongly fluctuating environments (Wang et al., 2020). Even if the population from the hotter/drier climate has a fitness advantage during an extreme event, it does not necessarily indicate that the associated phenotypes or allelic variation would increase in frequency within the native site. Distant populations may not be well adapted to other key abiotic and biotic selective agents within a native site (Bell, 2013). This maladaptation could manifest as a trade‐off to heat resistance, where populations with high survivorship during the heat wave may also have lower fecundity at the novel site relative to the native population.

In cases where heat waves cause population declines or extirpation, natural levels of gene flow between geographically proximate populations may allow recolonization of populations that experience intense mortality or provide an influx of genetic variation (i.e., “genetic rescue”) (Bell & Gonzalez, 2009; Fitzpatrick et al., 2016). This could be particularly important in environments with patchy habitats or in those that occur across steep environmental gradients, as geographically close populations may not experience equally extreme conditions as the focal population (Orr & Unckless, 2014). Assessing how nearby populations perform during an extreme event and the factors that cause heterogeneity in fitness may be as important as examining a focal population as these populations could provide an influx of individuals and genetic diversity to the focal population.

In this study, we examine how populations of a model species for ecological genetics, the common yellow monkeyflower (*Mimulus guttatus*; *syn. Erythranthe guttata*), perform during an extreme heat wave. Annual populations of *M. guttatus* occur throughout western North America in inland areas with ephemeral water supplies such as rock walls, seepy meadows, and flood plains (Wu et al., 2008). Annual plants germinate during spring rains or snow melt and senesce after producing seed during dry summers. The timing and length of the growing season vary dramatically across the range and there is considerable variation in the climates that different populations experience (Kooyers et al., 2015). Annual *M. guttatus* exhibits a wide range of phenotypic variation that allows for local adaptation to divergent environments (Friedman et al., 2015; Hall & Willis, 2006; Kooyers et al., 2019; Troth et al., 2018) and also has some of the highest levels of standing genetic variation across plant species (Colicchio et al., 2021; Puzey et al., 2017; Twyford et al., 2020; Vallejo‐Marín et al., 2021). These studies suggest that the genetic and phenotypic variation necessary to respond to selection due to an extreme event is likely present somewhere across this wide range.

Although *M. guttatus* is a common species unlikely to be threatened with extinction due to climate change, high‐elevation populations in the Central Oregon Cascades are at risk. These populations have the shortest growing season of all known annual *M. guttatus* populations, lasting only from early June to mid‐July (Kooyers et al., 2015). There is significant year‐to‐year variation in environmental conditions that can shift the growing season up to a month earlier (Kooyers et al., 2019; Troth et al., 2018). While populations maintain extremely high levels of polymorphism due to temporally fluctuating selection and fine‐grain heterogeneity in water availability within populations, these populations are experiencing the extremes of the historical climatic normal (Mojica et al., 2012; Nelson et al., 2018; Troth et al., 2018) and adaptation lags to changing conditions have already been documented (Kooyers et al., 2019).

Here we investigate how *M. guttatus* populations from throughout the range and within local populations in the Central Oregon Cascades perform during an extreme, but short 8‐day heat wave at the beginning of the growing season. Specifically, we use a common garden experiment to compare fitness from populations throughout the range of annual *M. guttatus* and we collect phenology and fitness data from nearby populations to better understand the metapopulation‐wide consequences of an extreme heat wave. We use these data to address the following questions: (1) How do local populations perform relative to more geographically distant populations during an extreme event? (2) Do extreme weather events favor populations whose historical environment more closely matches the extreme event? (3) Are the consequences of heat waves homogenous across a metapopulation?, and (4) If not, is heterogeneity predictable by variation in environmental characteristics between sites? We find that native populations performed poorly in our common garden, but some distant populations that historically encounter extreme heat more frequently have far higher fitness. Local populations also show considerable variation in responses to the heat wave with some populations avoiding negative fitness consequences entirely.

## 2 MATERIALS AND METHODS

### 2.1 Documenting an extreme event

During the second week of June 2019, we observed abnormally high temperatures and rapid dry‐down following snowmelt in our long‐term common garden and observation sites in the central Oregon Cascades. We took advantage of our existing infrastructure and ongoing experiments to examine the impact of this heat wave on *M. guttatus* populations. We quantified the magnitude of this heat wave by comparing climate in 2019 to historic averages. We downloaded monthly averages of minimum, average, and maximum temperatures as well as average precipitation and precipitation as snow for each year from 1980 to 2019 from ClimateWNA (Wang et al., 2016) for the Browder Ridge, Oregon common garden site (44.37348, −122.13055, Elev. = 1246 m asl). For more precise and frequent temperature observations during the growing season, daily summary data from 1980 to 2019 was downloaded for the closest NOAA weather station (Santiam Jct.: 44.44, −121.95, Elev. = 1140 m asl, 16 km from the garden) from NOAA's National Climatic Data Center.

### 2.2 Common garden study

**FIGURE 1:**
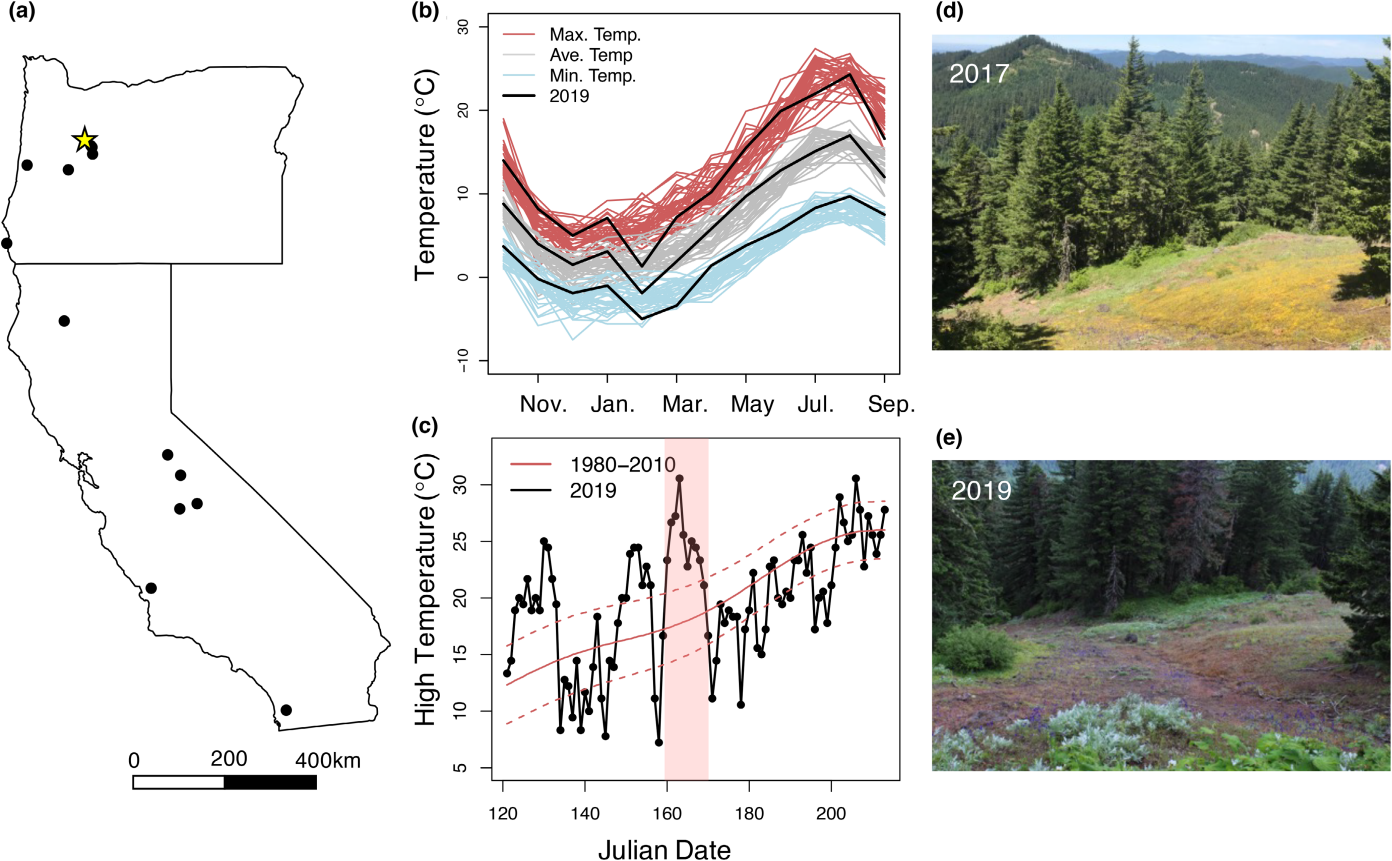
Locations of populations and description of weather patterns during the 2019 growing season. (a) Map of the populations used within the common garden experiments (black dots) as well as the location of the Broader Ridge common garden site (yellow star). (b) Annual climate patterns from 1980 to 2019 constructed from monthly averages of minimum (blue), average (gray), and maximum temperature (red). Black lines are data from the 2019 growing season. (c) Maximum daily temperatures during the 2019 growing season (black). The solid red line represents the historical average maximum temperatures (1980–2010) for each day, and the dashed lines represent 99% confidence intervals. The vertical red bar indicates days of extreme heat wave. (d, e) Photographs were taken in the exact same location at the nearby Iron Mountain population of *Mimulus guttatus* on June 29, 2017 and July 2, 2019.

To examine how the heat wave influenced relative patterns of adaptation, we leveraged a common garden field experiment that contained outbred lines from 11 populations spanning much of the range of annual *M. guttatus* at the Browder Ridge site (Figure 1a). Each outbred line was derived from seeds collected from 5 to 8 maternal families (Ave. 7.1). Latitude, longitude, and elevation of each site were taken at the time of collection and used to acquire climatic norms (1960–1990) for each population from ClimateWNA as well as haversine distances from each population to the Browder Ridge common garden. To generate outbred lines, maternal lines were grown for a single generation in a common garden greenhouse environment and crossed to another maternal line from the same population. Each maternal line acted as a pollen recipient in one cross and a pollen donor in a second cross.

We initiated the common garden by planting outbred line seeds in 2.25″ pots filled with Sunshine #1 (Sun Gro Horticulture) in unperforated 10″ × 20″ flats. We covered the flats with clear humidity domes, and cold‐stratified the seeds in the dark at 4°C. After 7 days of stratification, we moved the flats to the University of Oregon greenhouse with ambient light and temperature conditions. Plants were misted and germination was recorded daily. Following 7 days in the greenhouse, we removed humidity domes and plants were bottom‐watered as needed. After 14 days in the greenhouse, we randomized all pots into 12 blocks and transplanted seedlings directly into the field site. Microsite variation in water availability is high at this site with a natural population of *M. guttatus* spanning areas that dry out at different rates. We planted the blocks in locations that span this variation. Loss due to transplant shock has been minimal at this field site in past years (Colicchio, 2017; Kooyers et al., 2019; Troth et al., 2018). Timing matched the phenology of local populations. That is, all native plants were vegetative rosettes within a few weeks of germination.

We surveyed survival and flowering for each plant every other day. Survival was defined as having any living green tissue in leaves, stem or meristem (i.e., active chloroplast activity). No plant that was recorded as dead appeared later in the growing season as alive. To assess fecundity, we counted the number of flowers, collected all mature calyxes, and counted the seeds they contained. Below we report “inclusive number of flowers” as the total number of flowers where plants that did not survive or flower counted as zeros and “inclusive number of seeds” as the number of seeds produced where plants that did not produce seeds counted as zeros. We report both metrics because the number of flowers better includes fitness contributions through male fecundity while the number of seeds better represents female fecundity.

### 2.3 Assessing differences in fitness between populations in the common garden

We determined how the native population performed relative to other populations throughout the *M. guttatus* range using linear mixed models and generalized linear mixed models (LMMs and GLMMs) implemented with the *lmer()* and *glmer()* functions in the *lme4* v1.1‐27.1 package (Bates et al., 2014) in R v4.1.1 (Institute for Statistic Computing). Separate univariate models were constructed for five different fitness components (survival to flowering, number of flowers, number of seeds, inclusive number of flowers, and inclusive number of seeds) as the response variable. The number of seeds and the inclusive number of seeds were both log‐transformed to improve the fit of the below models. Each model had population as a fixed factor and line as a random factor. Additionally, variation among blocks in the garden was included as a random factor in both models. The GLMM assessing survival to flowering had a binomial error distribution with a logit link while LMMs were used for the number of flowers, number of seeds, inclusive number of flowers, and inclusive number of seeds (GLMMs using a Poisson family, and logit link had nearly identical results; Appendix 1: Table A1). Statistical significance of population was assessed via ANOVA using the *Anova()* function in the *car* v3.0‐12 package (Fox et al., 2013). We compared the native population to all other populations by calculating line means for each variable and conducting Dunnett's tests with BR1 as the focal population. Dunnett's tests were implemented using the *DescTools* v0.99.44 package. We note that our estimates of absolute fitness are likely elevated from natural populations as we transplanted seedlings to limit initial mortality.

We also investigated potential trade‐offs between survival of the heat wave and fecundity by comparing whether lines that had a higher chance of surviving the heat wave were more likely to have higher fecundity. We constructed linear models to examine the association between the probability of surviving until flowering for a given maternal line and the average number of flowers or average number of seeds that the maternal line produced. Models were implemented using the *lm()* function and the number of seeds was log‐transformed as above. Additional models with a logit transformation of the probability of surviving until flowering produced qualitatively identical results. A negative correlation between the probability of survival and fecundity is indicative of a trade‐off.

### 2.4 Fitness‐historical environment associations

We examined whether historic climatic differences among populations were associated with differences in fitness using a univariate GLMM approach with fitness variables as the response variables in independent models. All models included block, population, and line nested within the population as random variables and the environmental variable as a fixed factor. For each fitness variable, we ran separate GLMM for seven different environmental variables including geographic distance to the Browder Ridge common garden, mean annual temperature, annual heat moisture index, growing season start date, precipitation as snow, variance in spring maximum temperature, and variance in summer maximum temperature. Variances were calculated from maximum spring and summer temperatures from 1960 to 2021 extracted from ClimateWNA. These factors were chosen because they have all been identified as potential agents of selection for *M. guttatus* in past studies (Kooyers et al., 2015, 2019). Error distributions, links, and transformations are the same as described above. Statistical significance was determined by ANOVA as above.

### 2.5 Impact of the heat wave on natural populations

**FIGURE A1:**
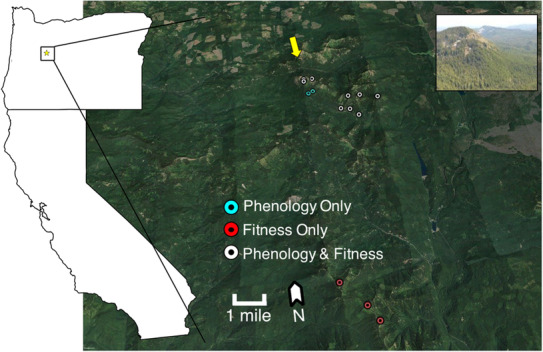
Map of Browder Ridge monkeyflower populations. White points correspond to populations used for phenology and fitness measurements within natural populations. Red points correspond to populations used for fitness and blue to populations used only for phenology measurements. Yellow arrow reflects the location and direction that the insert photo was taken.

We selected 11 natural populations distributed across an elevational gradient of ~600 m in a 10 km^2^ region of Browder Ridge to examine variation in soil moisture, survivorship, and phenology across the 2019 growing season (Appendix 2: Figure A1). In each population, we surveyed two 0.25m^2^ sampling grids (50 × 50 cm) every 7–14 days from snowmelt until population senescence. On each visit, we counted the number of vegetative, flowering, and senesced plants as well as measured volumetric water content with an SM150T soil moisture sensor (Dynamax). Grid locations were chosen within sites to encompass the natural variation within the site while only using areas with high concentrations of *M. guttatus* seedlings.

We examined how the number of individuals in each grid changed before, during, and after the heat wave to assess the mortality associated with the heat wave. To examine whether differences in soil moisture were driving differences in mortality between plots, we used an LMM to model whether the amount of mortality experienced in a grid during the heat wave was associated with the volumetric soil water content before the heat wave. Population was treated as a random factor in these models. Statistical significance of the fixed factors was assessed with lmerTest v3.1‐3 using Satterthwaite's degrees of freedom method (Kuznetsova et al., 2017). We further examined variation in survivorship, phenology, and volumetric water content throughout the growing season by modeling mortality, flowering, and soil moisture through time and calculating summary statistics for each grid (see Appendix 3 for further methodology and models). Summary statistics included critical survivorship date (when 50% of plants still survived in a plot), peak flowering date, and date when VWC fell below 20% for the first time.

### 2.6 Impacts of the heat wave on fecundity in natural populations

We examined the influence of the heat wave on fecundity using observational data collected at the end of the 2018 and 2019 growing seasons from 12 natural populations. Nine of these populations were the same ones as described above (Appendix 2: Figure A1). We chose plants in each population using two 7.5 m transects running through the center of each population. The same transect locations were used in 2018 and 2019. We identified the closest plant to the survey line every 15 cm along the transect and counted the number of flowers and seeds for each plant. We collected 100 plants/population when possible but did not make the full collection in every population in 2019 as three populations had very few plants producing seeds (HDM, OWC, RRM). For these populations, we collected <10% of the total individuals within the population. We modeled how the number of flowers and the number of seeds changed between years and populations using an LMM with a log of seed set as the response variable and Population, Year, and Population:Year interaction as independent variables. Statistical significance was determined using *Anova()* as above with a type III sum of squares. Because the interaction between population and year was significant, we examined population means to determine the direction and magnitude of differences in each individual population between years. We also evaluated whether the relative differences in flower or seed production between 2018 and 2019 that we observed between populations was associated with the environmental characteristics of the populations. We modeled associations between the difference in seed production between years and each of six different environmental characteristics (elevation, mean annual temperature, mean coldest month temperature, the beginning of the frost‐free period, precipitation as snow, and climate moisture deficit) using linear regressions implemented through the lm() function. Differences in seed production between years were calculated from population averages.

## 3 RESULTS

### 3.1 Early season heat wave decimates M. guttatus experimental garden

Climate patterns in 2019 in the Central Oregon Cascade Mountain Range closely resembled historic monthly normals for both temperature and precipitation (Figure 1b). However, a severe heat wave occurred approximately 2 weeks following snow melt at the Browder Ridge field site. Data from the nearest NOAA weather station indicate max temperatures over an 8‐day period were the hottest on record back to 1983 (Days 160–167; Figure 1c) and peaked at 30.4°C on Day 163 (June 12). The average maximum temperature during this stretch in 2019 exceeded the historic average maximum temperature by 8.5°C. In the Browder Ridge common garden, 89.0% of seedlings (438/492) died during the heat wave. There was a significant effect of block on survivorship (*χ* ^2^ = 141.1, *p* < .001). Survivorship in blocks ranged from 0% to 77.3% and only three blocks had >15% survivorship. Blocks where soil dried out later in the heat wave had higher survivorship suggesting that death was due to a combination of limited water availability and heat stress (A. Scharnagl, personal observation).

### 3.2 Native populations have low relative fitness in common garden

**FIGURE 2:**
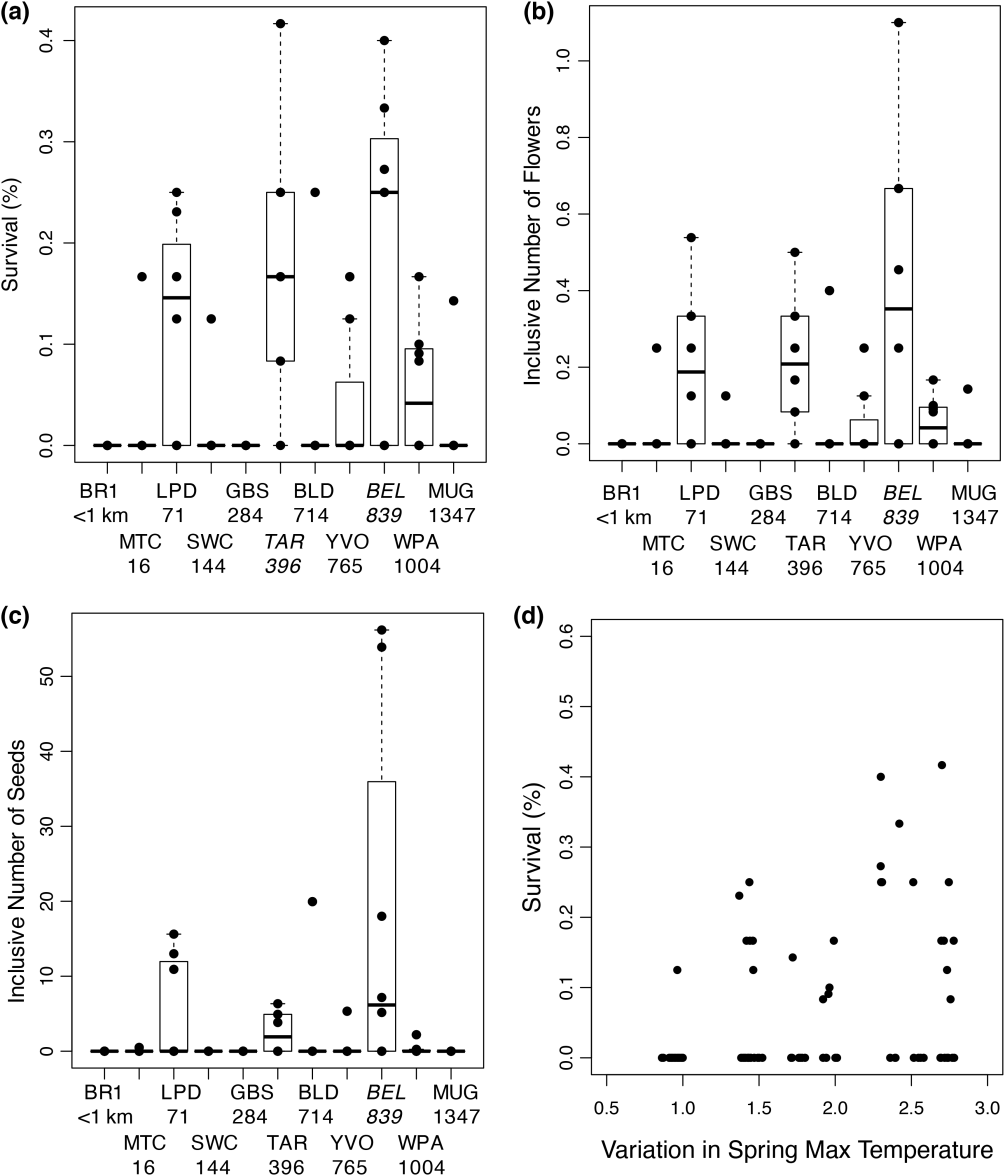
Variation in fitness between populations within the common garden experiment. Survivorship to flowering (a), inclusive number of flowers (b), and inclusive number of seeds (c) for each population within the common garden experiment. Points represent line averages. Values below population abbreviations are the distance from the Browder Ridge common garden site in kilometers. Italicized population names are the populations that significantly differ in survivorship from the BR1 population at *α* = .05 in a Dunnett test. (d) Association between survival to flowering in the common garden and the source population's variance in spring maximum temperature. Points represent the percentage of individuals that survived to flowering within a given line. Points are slightly jittered along the *x*‐axis in order to better see the number of lines per population with no survival.

There were significant differences in survivorship among populations planted in the common garden following the heat wave (*χ* ^2^ = 22.9, *p* = .01, Appendix 1: Table A1). Populations from locations geographically close to the common garden site with similar historical climate conditions had extremely low survivorship following the drought (Figure 2a, Appendix 4: Table A2). The BR1 population did not have a single individual survive the heat wave (0/21 seedlings; 0.38 km away) and the MTC population had only two individuals survive (2/37 seedlings; 15.6 km away). Instead, three more distant populations exhibited notably higher survivorship than the other populations. A nearby low‐elevation population (LPD, 71.2 km away) had 16.4% of seedlings survive the drought, a population from the Klamath Mountains (TAR, 396 km away) had 17.2% survival, and a population from the foothills of the Sierra Nevada Mountains (BEL, 839 km away) had 24.2% survival.

**FIGURE A2:**
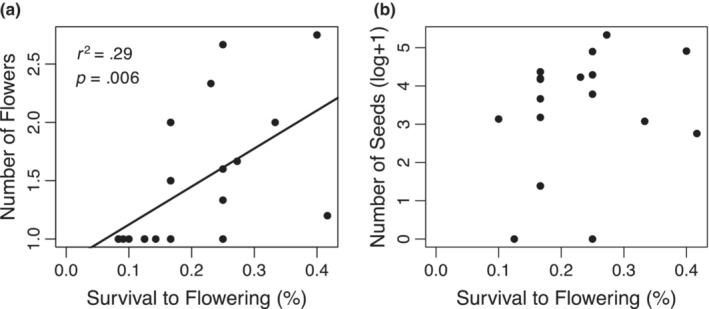
Scatterplots depicting relationships between fitness measures in the common garden study. Plots depict associations between survival to flowering and either inclusive number of flowers produced (a) or inclusive number of seeds produced (b) during the 2019 growing season. Each point represents an outbred line mean.

Following the heat wave, there were no significant differences in fecundity among populations (number of flowers: *F*_8,42.6_ = 1.8, *p* = .10; number of seeds: *F*_6,10.4_ = 1.4, *p* = .30, Appendix 1: Table A1) and metrics of fitness that include both survival and fecundity closely resembled survival models, with significant variation among populations (Inclusive Number of Flowers: *χ* ^2^ = 47.0, *p* < .0001; Inclusive Number of Seeds: *χ* ^2^ = 40.1, *p* = .0002; Figure 2). Of the four populations that produced >1 seed/plant on average (i.e. an approximation of replacement level), the closest population to the BR common garden site was a low‐elevation Oregon site (LPD) located 71.2 km away and the other three populations were from California. There was no evidence of a tradeoff between ability to survive the heat wave and fecundity following the heat wave. Lines that had higher survival during the drought produced more flowers than lines that had lower survival (*r* ^2^ = .29, *p* = .006; Appendix 5: Figure A2), but there was no relationship between viability and the number of seeds produced (*r* ^2^ = .07, *p* = .28, Appendix 5: Figure A2). Together these results suggest that local populations are less likely to survive and do not have a fecundity advantage over geographically distant populations following an early season heat wave.

### 3.3 Populations from more arid areas do not necessarily perform better

**FIGURE A3:**
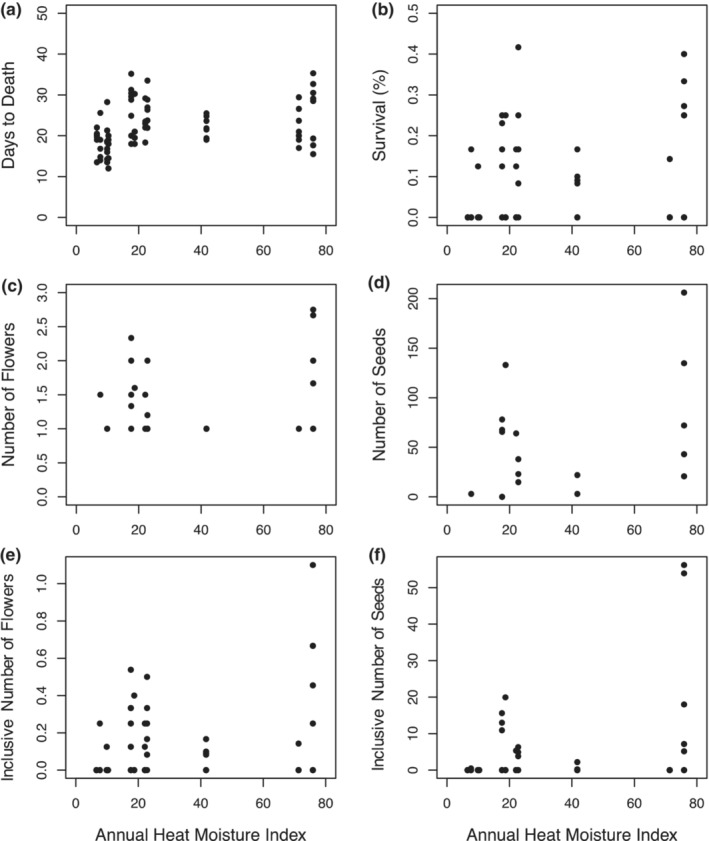
Relationships between fitness measures and Annual Heat Moisture Index (AHM) from the source population within the Browder Ridge common garden site. Points represent line averages or in the case of survival to flowering, the percentage individuals that survived to flowering within a given line. The only significant association is between inclusive number of seeds and AHM (D; *χ* ^2^ = 5.9, *p* = .01).

Although there were clear differences among populations in both survivorship following the heat wave and inclusive number of flowers and seeds, these differences are not tightly associated with the historical environments of the populations (Figure 2d, Appendices 6 and 7). The only variable even marginally associated with any fitness metric was variation in spring maximum temperature with survival (*χ* ^2^ = 3.6, *p* = .06; Figure 2d). The three populations that best survived the heat wave were from notably warmer and more arid climates than Browder Ridge but there are other populations from similarly warm/arid climates that had very low survivorship (Appendix 6: Figure A3). Likewise, neither the inclusive number of flowers nor seeds was associated with any geographic or historical climate predictor (Appendix 7: Table A3).

### 3.4 Widespread but variable mortality across a metapopulation

**FIGURE 3:**
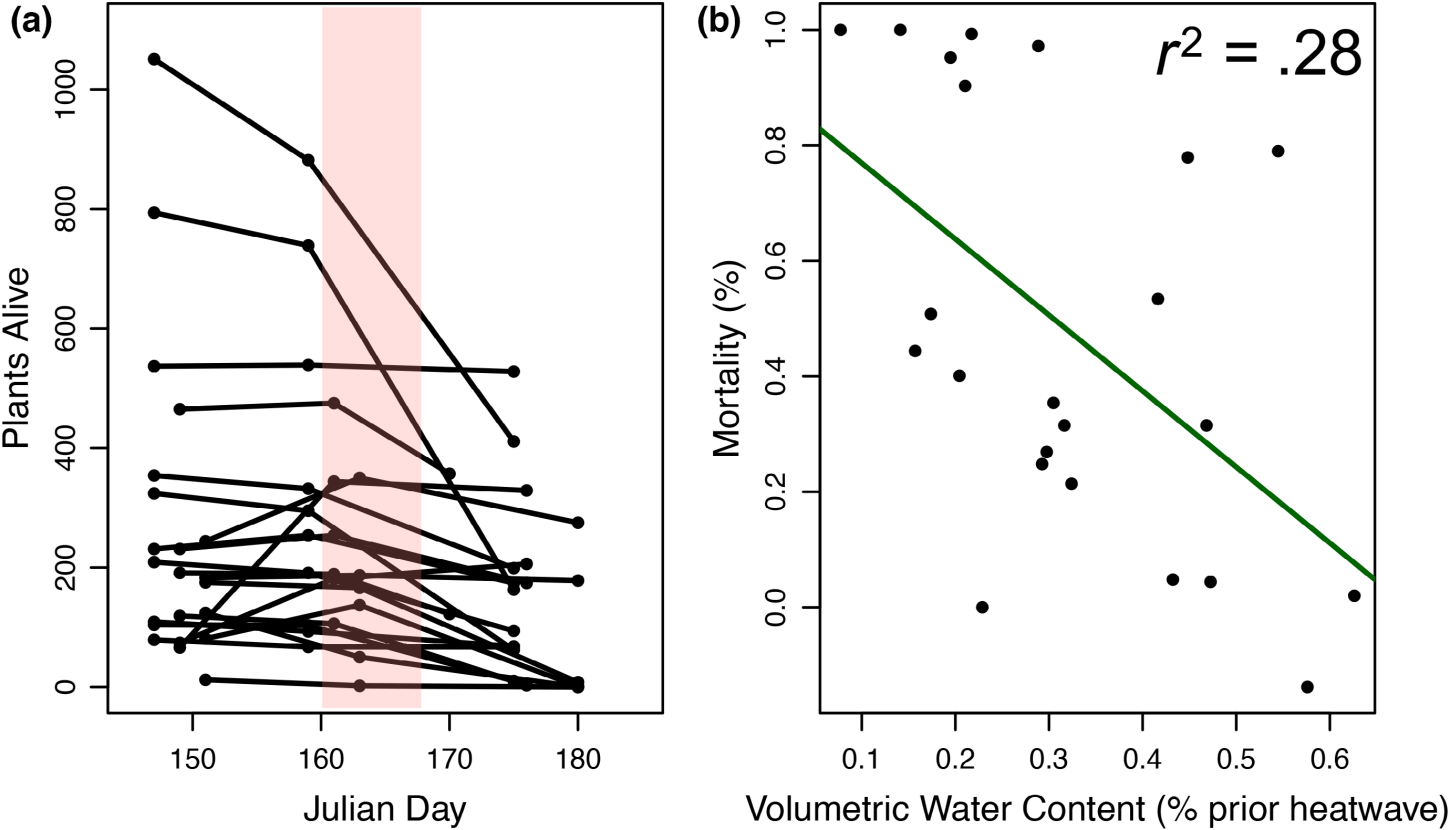
Responses across natural populations to the extreme heat wave. (a) The plot depicts the number of plants alive in each grid surveyed before, during, and after the heat wave (the heat wave occurred from days 160 to 167). (b) The relationship between mortality and soil moisture prior to the heat wave in each grid. The solid line is the regression line of best fit.

To better understand how this extreme heat wave could impact *M. guttatus* populations across a relatively small geographic region, we followed the survivorship and phenology of 11 populations (two 0.25 m^2^ sampling grids per population) at locations across Browder Ridge. Indeed, the heat wave *did* cause mortality for every population surveyed except one (MBR). The number of individuals in 23 of 24 grids declined during the heat wave (Figure 3a). While the number of individuals per grid increased by 10% on average in the week prior to the heat wave, the average number of individuals in a grid declined by 50.4% (SD: 30.96%) between the pre‐ and post‐heat wave sampling points (Appendix 8: Table A4). One population (HDM) had no individuals from either grid survive the heat wave. Other populations had extreme differences in survivorship between grids within a single population. For instance, one grid in BR1 had only 2% mortality during the heat wave while the other grid, located only ~1.5 m away, had 53.4% mortality. There was no relationship between elevation or other coarse environmental factors and survivorship in each grid during the heat wave (Appendix 9: Table A5). This lack of a pattern suggests small‐scale microclimatic variation found within a population is most predictive of survivorship.

### 3.5 Soil moisture is associated with survivorship

**FIGURE A4:**
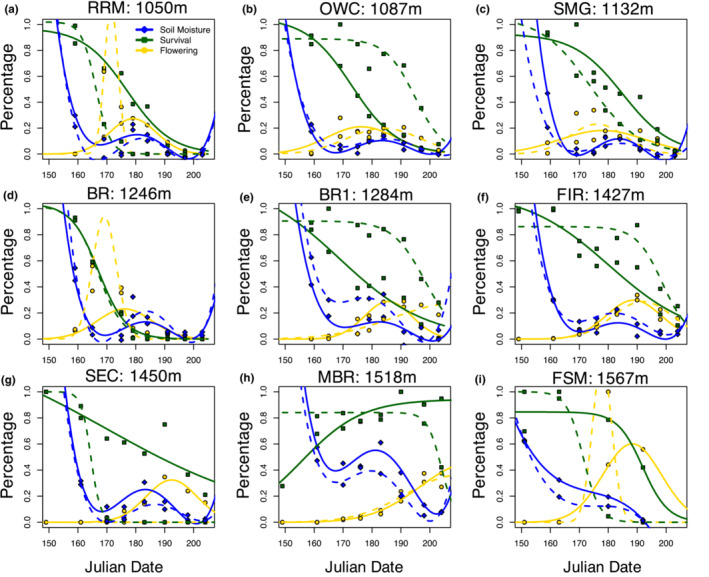
Survivorship and phenology during the 2019 growing season for Browder Ridge monkeyflower populations. Each scatterplot (a–i) depicts volumetric water content (blue), proportion of the population surviving (green), and proportion of the population flowering (yellow) during the growing season from a single population. Three letter abbreviations and elevations are given above each graph. Lines represent inferred models for each of the two grids within a population and different grids are represented by dashed vs solid lines.

**FIGURE A5:**
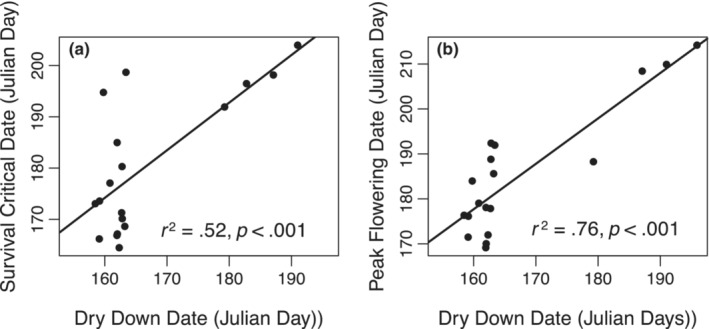
Associations between dry‐down dates and population phenology for Browder Ridge monkeyflower populations. Scatterplots depict associations between dry down date and either critical survival date (a) or flowering peak date (b). Dry down date was defined as the predicted day where volumetric water content drops below 0.2. Critical survival date was defined as the inflection point on a survival time series model. Peak flowering date was defined as the predicted day of peak flowering from fitted models.

Since *M. guttatus* populations are characterized by having ephemeral supplies of water, we examined how soil moisture, survivorship, and phenology varied within and between natural populations as a potential key factor for understanding responses to the heat wave. Nearly all sampling grids dropped below 20% VWC during the heat wave, although several populations, particularly at higher elevations, returned above 20% later in the growing season (Appendix 10: Figure A4). There was a strong association between soil moisture before the heat wave and the mortality during the heat wave where grids with lower VWC before the heat wave had higher mortality (*χ* ^2^ = 9.1, *p* = .003; Figure 3b)—that is grids that dried down earlier had plants die earlier. Peak flowering occurred after critical survivorship dates in 76% of grids indicating that most plants died before flowering. There were substantial differences in peak flowering among populations with peak flowering occurring later in populations with later dry‐down dates (Appendix 11: Figure A5). Combined, these data suggest that survival and phenology in these populations are strongly associated with variation in soil moisture rather than the actual heat stress associated with the heat wave.

### 3.6 Heterogeneous responses of the heat wave across the metapopulation

**FIGURE 4:**
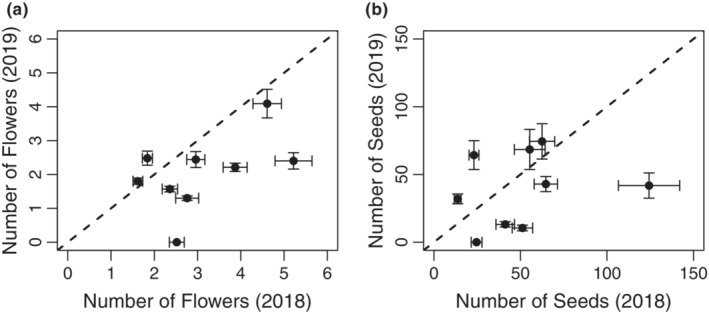
Comparisons of fitness in Browder Ridge monkeyflower populations in 2018 versus 2019. Scatterplots depicting the number of flowers (a) and number of seeds (b) in 2018 versus 2019. Error bars represent standard error.

We compared fecundity measures from 12 populations between 2018 (a relatively normal year) and 2019 (an extreme heat wave early in the growing season). There was a significant population‐by‐year effect on both number of flowers (*F*_7,1484_ = 12.3, *p* < .0001) and number of seeds (*F*_7,1476_ = 21.3, *p* < .0001). The majority of natural populations had lower fecundity during the year of the heat wave (2019). In the populations that did worse in 2019 than in 2018, the number of flowers and the number of seeds were reduced by an average of 36% and 41%, respectively (Figure 4, Appendix 8: Table A4). However, some populations had higher fecundity during the heat wave year. Two populations produced more flowers in 2019 than in 2018 (Figure 4; FIR and SEC) and four populations produced more seeds (Figure 4; FIR, SEC, OWC, SMG). Differences in fecundity between 2018 and 2019 were not associated with elevation, distance between populations, or any other environmental correlate that we examined (Appendix 12: Table A6). These data suggest that although the entire metapopulation experienced a heat wave, there was a large variation in how the heat wave impacted monkeyflower populations that is not predictable by the historic climate of a population.

## 4 DISCUSSION

The demographic consequences of extreme events, such as heat waves, are rarely explored, but such results are increasingly important in a changing climate. Our study provides a comprehensive examination of the heterogeneous consequences of a short but extreme heat wave for local and geographically distant populations of *M. guttatus*. Specifically, we document that Central Oregon populations experienced an extreme heat wave where high temperatures exceeded previous records. Plants from populations located near our common garden site were not well adapted to survive this event and those that did produced very few seeds. However, several of the more distant populations were better able to survive and produce seeds. While these surviving populations did come from areas with greater variation in spring max temperature or historically lower annual heat‐moisture indexes, other populations from similar areas did not have higher fitness. The majority of natural populations near our common garden had lower fitness in the year of the heat wave relative to the previous year and one population had no individuals reproduce. However, some native populations did not experience the same degree of mortality and this variation was strongly associated with soil moisture levels. Together these results suggest that, even though some populations in the metapopulation could exhibit substantial decline or even extirpation, other nearby populations may not experience conditions as harsh and could act as source populations for highly‐impacted populations. Below, we discuss the implications of our results and compare them to other studies of population dynamics following extreme events.

### 4.1 Mortality within a heat wave

Our results indicate mortality during a heat wave can be extreme and may have long‐lasting consequences for a population. While our heat wave was relatively short, we observed high mortality within our common garden experiment (~90% of plants died) as the heat wave occurred just as experimental plants were finishing establishing (transplanted 7 days prior to the start of heat wave). While this could be considered mortality due to establishment shock, we suggest this is relatively unlikely given our observations in nearby natural populations. That is 11 of the 12 natural populations had drastic declines in soil moisture and high mortality during this period (Figure 3a). Rates of mortality in natural populations pre‐ and post‐heat wave averaged 50.3% (SD 36.3%, range, 4.4%–100%). These rates are higher than many other studies documenting natural heat waves and/or droughts in plant populations despite being a very short heat wave in a relatively normal year (Allen et al., 2010; Harrison & LaForgia, 2019; Orsenigo et al., 2014; Thomson et al., 2018, but see Marrero‐Gómez et al., 2007). For instance, estimates of mortality from 17 plant species during a 2‐year heat wave/drought in Western Australia averaged 26.0% mortality (SD 24.0%, range 0%–71%) (Ruthrof et al., 2018). However, the majority of previous studies of heat waves may not be comparable as they have largely focused on longer events that are also associated with drought (Batllori et al., 2020) or examined longer‐lived species (Mueller et al., 2005; Ruthrof et al., 2018). Basic data on the survival of herbaceous plants to extreme conditions in nature is a necessity for predicting future plant population dynamics but is currently in short supply.

### 4.2 Historical aridity does not accurately predict heat wave survival

While mortality was high within both our common garden and natural populations, the fact that a minority of plants did survive and reproduce suggests there may be traits that facilitate survival. We expect populations from areas with historic climates that more closely match the extreme event to have higher fitness than native populations that rarely encounter such conditions – that is, an adaptation lag (Anderson & Wadgymar, 2020; Kooyers et al., 2019; Wilczek et al., 2014). Alternatively, these distant and potentially preadapted populations could perform as badly or even worse than native populations because the distant populations may be poorly adapted to other agents of selection within the native population (Scharnagl et al., 2023; Wadgymar et al., 2017) or may have a tolerance strategy not well suited to short but extreme events (Campbell‐Staton et al., 2021). Our results fall somewhere between these two extremes—local populations had high mortality and low fitness—and some distant populations from hotter drier areas had higher fitness (Figure 2, Appendix 4: Table A2). However, several populations that we assumed would do well under heat‐stressed conditions (i.e. populations from southern California and the high elevations in the Sierra Nevada) also had very low fitness.

A key question is why some populations that are historically warmer and drier did not have improved survival of the heat wave. We suggest that variation in traits among the populations likely provides some explanation. For instance, one of the populations with relatively high survival during the drought, LPD, is a low‐elevation site located only 71.2 km from the common garden site. This site does not typically experience the higher temperatures and aridity of most of the California populations but may be better adapted to the Cascades through having more similar growing season timing or biotic interactions. Indeed, low‐elevation Cascades plants do have more similar photoperiod requirements for flowering and chemical defense arsenals to high‐elevation Cascades populations than the California populations used within this study (Kooyers et al., 2015, 2017; Scharnagl et al., 2023). Notably, there are also large differences in magnitude and traits involved in plastic responses to dry‐down conditions for the different populations used within this experiment, including differences in responses among California populations that could explain differential mortality (FitzPatrick et al., 2023). Future work should aim to link variation in trait variation and physiology to fitness consequences during extreme events.

One clear conclusion from our results is that the relative fitness patterns found in the heat wave‐associated growing season (2019) do not match previous patterns found at the same location. We conducted a similar common garden experiment at the same site with many of the same populations in 2014 (Kooyers et al., 2019). The 2014 growing season was one of the earliest growing seasons on record and, as in the 2019 growing season, some populations from California had higher fitness than native Oregon populations. However, a low‐elevation Sierra Nevada population that performed very poorly in 2014 (BEL) had the highest fitness of any population in 2019 and the high‐elevation Sierra Nevada populations that did well in 2014 had relatively modest fitness in the 2019 season (Figure 2, Appendix 4: Table A2). This difference is likely due to the nuanced changes in selection pressures between years. In 2014, abnormally high spring temperatures led to the growing season starting weeks earlier than normal, but precipitation was relatively normal throughout the year. In 2019, spring temperatures and snowfall were near average leading to a relatively normal growing season start date prior to the early season heat wave. Thus, in 2014, populations able to take advantage of the earlier‐than‐normal growing season were presumably favored while, in 2019, plants able to survive a high heat and low water climatic event as seedlings were likely favored. Temporal heterogeneity in selection pressures in these populations has been widely documented previously (Mojica et al., 2012; Troth et al., 2018), but this study demonstrates the fluctuations in the environment are substantial enough to enable populations with the lowest relative fitness in 1 year to have the highest relative fitness another year. These results suggest predicting a population's tolerance to an extreme event is not as simple as examining historic environmental variation in the selective pressure under question.

### 4.3 Metapopulations experience variation in the consequences of extreme events

Here we report that the heat wave had severe consequences on natural populations near the common garden site including complete mortality within one population and >50% mortality prior to flowering in several other populations (Figure 3a). Our results suggest that the most important factor for predicting mortality during the heat wave was the amount of nearby soil moisture present prior to the heat wave (Figure 3b). This suggests that water rather than heat may have been the limiting factor during this heat wave. The consequences of this heat wave extended to differences in fecundity across most populations, with lower numbers of flowers and seeds produced per plant than in a more normal year (Figure 4). We hypothesize that the link between mortality and fecundity stems from either the delayed growth of seedlings following the heat wave or the selective mortality of smaller seedlings or rapidly reproducing plants during the heat wave. These hypotheses stem from observations of delayed phenology relative to other years within these populations (Appendix 10: Figure A4; N. Kooyers, personal observation). However, we cannot rule out altered interactions with pollinators or herbivores (Walters et al., 2022).

While the amount of mortality seems like a relatively dire result, populations seldom exist in isolation and nearby populations may influence focal population dynamics both during and following an extreme event. Importantly, nearby populations may not experience the same severe selective conditions that a focal population receives and thus can act as a source for new migrants and replenish genetic variation following an extreme event through dispersal or gene flow (Fitzpatrick et al., 2016; Whiteley et al., 2015). We suggest the heterogeneity we observe in mortality within and between populations mitigates the potential for complete extinction within the metapopulation due to a single extreme event and could allow for recolonization of extirpated sites. Gene flow between populations is likely high as there is very limited population structure between these populations (Colicchio et al., 2021). Recolonization has been frequently observed in other monkeyflower populations. For instance, over 25 years of observing 39 perennial populations of *M. guttatus* in the Wasatch mountains of Utah, there were 54 population disappearances and 34 reappearances that likely stemmed from seeds dispersing down rivers from upstream populations (Vickery, 1999).

However, in our system, gene flow from other populations may not be necessary for an influx of individuals or genetic variation as much of the variation in heat wave‐associated mortality was contained between grids within populations. The most important factor in predicting mortality was the soil moisture prior to the heat wave (Figure 3b), and there was substantial variation within populations in soil moisture. The highest elevation population (HOV) is a perfect example of this heterogeneity within a population: while one grid still had trace amounts of snow during the beginning of the heat wave and had nearly no mortality (4.8% dead), the other grid dried down during the heat wave and had nearly complete mortality (99.3% dead). Thus, despite very high mortality within different microclimates within a population, dispersal within populations could allow for rapid recovery from extreme events.

For populations that experience high mortality, an equally viable solution is the presence of a seed bank (Kalisz & McPeek, 1993; Walck et al., 2011). Seed banks in *M. guttatus* have long been hypothesized (Vickery, 1999), and our study provides additional evidence. We followed the ‘extirpated’ population during the heat wave for the next 2 years (HDM). While there was no germination the following year (2020), there were a limited number of germinants in 2021 (30–40 individuals, S. Innes, personal observation). The remote nature of this population and the number of germinants suggests that these germinants came from the seed bank rather than from dispersal. Our study highlights the difficulty in predicting species responses to extreme events. Detecting the risk of extirpation for other species requires determining how the particular event impacts the environment, how variation in the environment corresponds with variation in mortality, and accounting for reestablishment from the seed bank, dispersal, and gene flow.

### 4.4 Long‐term consequences of extreme heat waves

Long‐term survival in a changing climate may require more than just the resurrection of populations from the seed bank or genetic rescue from other nearby populations. The heterogeneity in mortality from the heat wave across populations suggests our metapopulation is a ripe environment for the rapid evolution of heat and drought‐resistance strategies (Grant et al., 2017). Such evolution to extreme events has been described in numerous other systems e.g. (Donihue et al., 2020; Franks et al., 2007; Grant & Grant, 1993) and can have long‐term consequences for the populations (Grant & Grant, 1993). Given the high levels of genetic and phenotypic variation present in our monkeyflower populations (Colicchio et al., 2021; Puzey et al., 2017) and a high degree of fine‐grain spatial and temporal heterogeneity in environmental factors that have promoted balancing selection (Troth et al., 2018), heat or drought‐resistance related alleles may already be present within certain populations at low frequencies. These populations also exhibit a very limited population structure which suggests that gene flow is likely high (Colicchio et al., 2021). Thus, an adaptive variant that evolves in one population will likely spread to other nearby populations relatively quickly.

In conclusion, this study suggests that extreme heat waves can cause drastic declines in native populations, but such mortality may be ameliorated by micro‐environmental variation, seed banks, and potential genetic rescue stemming from nearby populations. While this result is optimistic, we caution that survival of a single short‐term extreme event is not necessarily predictive when extreme events become normal.

## AUTHOR CONTRIBUTIONS

**Laura M. McDonald:** Conceptualization (supporting); formal analysis (supporting); investigation (equal); writing – review and editing (equal). **Anna Scharnagl:** Conceptualization (supporting); investigation (equal); writing – review and editing (equal). **Andrea K. Turcu:** Investigation (equal); writing – review and editing (equal). **Courtney M. Patterson:** Investigation (equal); writing – review and editing (equal). **Nicholas J. Kooyers:** Conceptualization (lead); formal analysis (lead); writing – original draft (lead); writing – review and editing (equal).

## FUNDING INFORMATION

Funding for this research came from the University of Louisiana, Lafayette and NSF grant DEB‐2045643 to NJK.

## CONFLICT OF INTEREST STATEMENT

The authors declare no competing interests that may have influenced this manuscript.

## Data Availability

All data sets from this manuscript have been uploaded to Dryad and can be found at this link: https://doi.org/10.5061/dryad.b5mkkwhh7.

## APPENDIX 1

**TABLE A1:** Summary of ANOVA results examining effects of population on survival within the common garden.

Response variable	Model: family (Link)	Fixed factor	*χ* ^2^	df	*p*
Survival to flowering	GLMM: Binomial (Logit)	Population	22.9	10	.011
Number of flowers	LMM	Population	14.3	8	.072
Number of seeds	LMM	Population	8.9	6	.178
Inclusive number of flowers	LMM	Population	47.0	10	<.001
Inclusive number of seeds	LMM	Population	40.1	10	<.001
Inclusive number of flowers	GLMM:Poisson (Logit)	Population	41.4	10	<.001
Inclusive number of seeds	GLMM:Poisson (Logit)	Population	20.5	10	.025

*Note*: Inclusive number of flowers of flowers or seeds refers to number of flowers or seeds with any individual producing no flowers or seeds having a value of zero. Number of seeds and inclusive number of seeds was log+1 transformed for all LMM.

Abbreviations: GLMM, Generalized Linear Mixed Model; LMM, Linear Mixed Model.

## APPENDIX 3 Survivorship and phenology extended methodology and modeling

### 3.1

These methods describe how we modeled mortality, phenology and soil moisture through growing season and calculated summary statistics for each grid within each population. We assessed the proportion of plants surviving at a given time point by dividing the number of plants alive by the maximum census within the sampling grid. At some high elevation sites, we started censusing right after snowmelt when there were no germinates at first sampling, thus the maximum census size occurred at the second or third sampling point. To examine how survival curves differed within and among populations we used non‐linear least square regression to fit logistic curves implemented with the *SSlogis()* function from the *stats* v3.6.2 package. To compare between grids and populations, we used this model to estimate when 50% survival occurred during the growing season (termed critical survival date). We also estimated the end of the growing season by calculating when survivorship was 5% of its maximum value. To assess flowering phenology, we divided the number of plants flowering in a grid at a given date by the total number of plants that had flowered throughout the experiment within that sampling grid. This measure is flawed as the same plant may be recounted as flowering multiple times, however the relative phenology comparison between plots is still informative for our purposes. To compare within and between populations, we fit Gaussian curves to phenology data and optimized the fit via the *optim()* function to minimize the sum of squared residuals. We used this model to estimate when flowering began in each grid (i.e., when 5% of plants had flowered) and when flowering peaked in each plot. To model how soil moisture changed throughout the growing season, we fit fourth‐order polynomial models using the *lm()* function. The fourth‐order model was selected because it best accounted for late season increases in soil moisture that improved fitness in some populations. To compare patterns of dry down between populations, we calculated the Julian day when volumetric water content (VWC) first reached 0.2 using fitted polynomial models. This value was selected based on previous dry‐down experiments with *M. guttatus* in controlled conditions (N. Kooyers, personal observation).

## APPENDIX 4

**TABLE A2:** Fitness summary statistics for populations within the common garden experiment.

Pop.	*N*	Lat.	Elev. (m)	Dist. (km)	AHM	Survival to flower	Flowers Ave. (SD)	Seeds Ave. (SD)	Inc. number of flowers	Inc. number of seeds
									Ave. (SD)	Ave. (SD)
BR1	8	44.37	1269	0.4	6.6	0 (0)	NA	NA	0 (0)	0 (0)
MTC	5	44.23	1400	15.6	7.7	0.03 (0.07)	1.5 (NA)	3 (NA)	0.05 (0.11)	0.1 (0.22)
LPD	8	43.92	277	71.2	17.6	0.12 (0.1)	1.67 (0.51)	42 (39)	0.2 (0.2)	4.94 (6.94)
SWC	8	44.01	204	143.6	9.9	0.02 (0.04)	NA	NA	0.02 (0.04)	0 (0)
GBS	6	42.42	93	284.3	10.3	0 (0)	NA	NA	0 (0)	0 (0)
TAR	6	40.85	778	396.1	22.8	0.18 (0.14)	1.06 (0.83)	25 (12)	0.22 (0.18)	2.51 (2.87)
BLD	5	38.14	1733	714.1	18.7	0.05 (0.11)	2 (NA)	133 (NA)	0.08 (0.18)	3.99 (8.92)
YVO	8	37.72	1495	765.9	22.1	0.04 (0.07)	1.5(NA)	64 (NA)	0.05 (0.09)	0.67 (1.89)
BEL	8	37.04	196	839.2	75.9	0.19 (0.16)	2.08 (0.74)	95 (75)	0.39 (0.4)	17.55 (23.9)
WPA	8	35.43	377	1003.8	41.7	0.06 (0.06)	1 (0)	13 (13)	0.06 (0.06)	0.31 (0.77)
MUG	8	32.96	331	1346.9	71.3	0.02 (0.05)	NA	NA	0.02 (0.05)	0 (0)

*Note*: Fitness measures are all given in the format: mean (standard deviation). Inc. number of flowers or seeds refer to the number of flowers or seeds respectively and include all plants that did not survive to flowering.

Abbreviations: AHM, Annual Heat Moisture Index; Dist., Distance from the common garden site; Elev., elevation; *N*, Number of maternal lines per population; Pop., Population.

## APPENDIX 7

**TABLE A3:** Model results examining environmental associations with fitness metrics for populations within the common garden.

Response variable	Model	Independent variable	*χ* ^2^	df	*p*‐Values
Distance to common garden	GLMM: Binomial (Logit)	Survival to flowering	0.0135	1	.9075
Annual heat moisture index	GLMM: Binomial (Logit)	Survival to flowering	0.4303	1	.5119
Mean annual temperature	GLMM: Binomial (Logit)	Survival to flowering	0.202	1	.6531
Beginning of the frost free period	GLMM: Binomial (Logit)	Survival to flowering	0.0621	1	.8032
Climate moisture deficit	GLMM: Binomial (Logit)	Survival to flowering	0.9858	1	.3208
Variance in spring max temperature	GLMM: Binomial (Logit)	Survival to flowering	3.5907	1	.0581
Variance in summer max temperature	GLMM: Binomial (Logit)	Survival to flowering	0.8803	1	.3481
Distance to common garden	LMM	Inclusive number of flowers	0.0022	1	.9629
Annual heat moisture index	LMM	Inclusive number of flowers	1.1446	1	.2847
Mean annual temperature	LMM	Inclusive number of flowers	0.5169	1	.4722
Beginning of the frost free period	LMM	Inclusive number of flowers	0.1221	1	.7268
Climate moisture deficit	LMM	Inclusive number of flowers	0.7655	1	.3816
Variance in spring max temperature	LMM	Inclusive number of flowers	1.2113	1	.2711
Variance in summer max temperature	LMM	Inclusive number of flowers	0.205	1	.6508
Distance to common garden	LMM	Inclusive number of seeds	0.0403	1	.841
Annual heat moisture index	LMM	Inclusive number of seeds	1.7181	1	.1899
Mean annual temperature	LMM	Inclusive number of seeds	0.8435	1	.3584
Beginning of the frost free period	LMM	Inclusive number of seeds	0.2657	1	.6062
Climate moisture deficit	LMM	Inclusive number of seeds	1.1512	1	.2833
Variance in spring max temperature	LMM	Inclusive number of seeds	1.3747	1	.241
Variance in summer max temperature	LMM	Inclusive number of seeds	0.1126	1	.7372

*Note*: Inclusive number of flowers or seeds refer to the number of flowers or seeds, respectively, and include all plants that did not survive to flowering. Female fitness was log+1 transformed for all models.

Abbreviations: GLMM, Generalized Linear Mixed Model; LMM, Linear Mixed Model.

## APPENDIX 8

**TABLE A4:** Mortality and phenology of natural populations near the common garden site.

Site	Elev. (m)	Seeds 2018	Seeds 2019	Flowers 2018	Flowers 2019	Grid	Alive (initial)	Alive (before)	Alive (after)	Mortality rate	VWC date (<0.2)	Surv. date (50% Surv.)	Flowering peak date
		Ave. (SD)	Ave. (SD)	Ave. (SD)	Ave. (SD)								
RRM	1050	124.3 (176.9)	41.8 (64.9)	5.2 (4.3)	2.4 (1.7)	A	109	93	68	0.27	160.8	177.1	179
						B	104	103	10	0.9	159.1	166.2	171.5
OWC	1087	55.4 (88.6)	68.4 (104.5)	3 (2.1)	2.4 (1.7)	A	209	191	94	0.51	158.5	173.1	176.4
						B	79	67	67	0	159.8	194.8	184
SMG	1132	62.5 (73.1)	74.4 (130.3)	4.6 (3.3)	4.1 (4.2)	A	231	254	174	0.32	162	185	178.1
						B	354	332	199	0.4	159.2	173.6	176.1
BR	1246	–	–	–	–	A	794	739	163	0.78	162	167.2	170.1
						B	324	295	62	0.79	162	166.9	169.2
BR1	1284	–	–	–	–	A	1051	882	411	0.53	163.2	168.6	185.6
						B	537	539	528	0.02	187.1	198.2	208.4
FIR	1427	13.7 (19.7)	31.9 (36)	1.6 (1.1)	1.8 (0.9)	A	191	189	122	0.35	162.8	180.3	188.8
						B	465	475	357	0.25	163.4	198.7	191.9
SEC	1450	23.1 (28.3)	64.3 (106.7)	1.8 (1.2)	2.5 (2.1)	A	231	254	174	0.32	162.8	170.1	192.4
						B	119	106	3	0.97	162.3	164.5	172
HDM	1465	24.6 (31.1)	–	2.5 (1.7)	–	A	124	50	0	1	156.3	–	–
						B	12	2	0	1	154.4	–	–
FSM	1535	51.2 (59.4)	10.5 (20.7)	2.4 (1.8)	1.6 (0.8)	A	244	350	275	0.21	179.3	191.9	188.3
						B	175	166	8	0.95	162.7	171.3	177.9
MBR	1518	41.2 (53.7)	13.1 (19.8)	2.8 (2.7)	1.3 (0.7)	A	209	191	94	0.51	195.9	–	214.2
						B	66	344	329	−0.04	191	204	209.9
HOV	1567	64.7 (67.3)	42.9 (55)	3.9 (2.8)	2.2 (1.2)	A	183	187	178	0.05	182.8	–	–
						B	81	137	1	0.99	165.2	–	–

*Note*: Dashes indicate that models were not run because either there was no survival following the heatwave or because phenology surveys ended before substantial decline of plants and flowering within a grid. All dates are Julian dates.

## APPENDIX 9

**TABLE A5:** Associations between phenology and environment variables within natural populations.

Response variable	Independent variable	Sum of squares	Mean square	Numerator df	Denominator df	*F*‐Value	*p*‐Value
Dry down date	Elevation	154.9	154.9	1	9	2.85	.126
Dry down date	MAT	251.3	251.3	1	8.9966	4.63	.06
Dry down date	MCMT	330.4	330.4	1	9	6.08	.036
Dry down date	bFFP	45.1	45.1	1	9	0.83	.386
Dry down date	PAS	267	267	1	9	4.92	.054
Critical survival date	Elevation	213.4	213.4	1	7.4632	1.36	.279
Critical survival date	MAT	118.8	118.8	1	7.4655	0.75	.412
Critical survival date	MCMT	139.6	139.6	1	7.871	0.89	.372
Critical survival date	bFFP	22.1	22.1	1	5.6088	0.13	.729
Critical survival date	PAS	173.5	173.5	1	8.2698	1.12	.32
Flowering peak	Elevation	219.9	219.9	1	7	3.32	.111
Flowering peak	MAT	206.6	206.6	1	7	3.12	.121
Flowering peak	MCMT	192.6	192.6	1	7	2.91	.132
Flowering peak	bFFP	10.6	10.6	1	7	0.16	.701
Flowering peak	PAS	212.3	212.3	1	7	3.21	.117

*Note*: Dry down date refers to the date that a grid registered a VWC of < 0.2. Critical Survival Date is the Julian day of the year with 50% of plants remaining. Flowering peak is the model estimate of Julian date with the highest frequency of plants flowering. Each response variable was derived from the corresponding models rather than actual observation dates. Associations between response variables and environmental predictor variables were determined with linear mixed models as described in the text.

Abbreviations: bFFP, Beginning of the Frost Free Period; MAT, Mean Annual Temperature; MCMT, Mean Coldest Month Temperature; PAS, Precipitation as Snow.

## APPENDIX 12

**TABLE A6:** Associations between natural population fecundity in 2018 and 2019 with environmental characteristics.

Response variable	Environmental predictor	*r* ^2^	*F*‐Value	df	*p*‐Value
Difference in average seed production	Elevation	.04	0.38	1,10	.55
Difference in average seed production	MAT	.03	0.35	1,10	.57
Difference in average seed production	MCMT	.03	0.35	1,10	.57
Difference in average seed production	bFFP	.03	0.26	1,10	.62
Difference in average seed production	PAS	.02	0.24	1,10	.64
Difference in average seed production	CMD	.11	1.28	1,10	.28
Difference in average flower production	Elevation	.09	1.03	1,10	.34
Difference in average flower production	MAT	.06	0.64	1,10	.44
Difference in average flower production	MCMT	.03	0.34	1,10	.57
Difference in average flower production	bFFP	.05	0.49	1,10	.5
Difference in average flower production	PAS	.01	0.12	1,10	.74
Difference in average flower production	CMD	.16	1.85	1,10	.2

*Note*: Differences in seed and flower production between 2018 and 2019 were calculated from population averages.

Abbreviations: bFFP, Beginning of the Frost Free Period; CMD, Climate Moisture Deficit; MAT, Mean Annual Temperature; MCMT, Mean Coldest Month Temperature; PAS, Precipitation as Snow.
